# CircTP63 promotes hepatocellular carcinoma progression by sponging miR-155-5p and upregulating ZBTB18

**DOI:** 10.1186/s12935-021-01753-x

**Published:** 2021-03-08

**Authors:** Jiantao Wang, Jinbiao Che

**Affiliations:** 1 Department of Hepatobiliary Surgery, Yantaishan Hospital, Jiefang Road, Zhifu District, Yantai, 264000 Shandong Province China; 2 Department of Gastroenterology, The Affiliated Yantai Yuhuangding Hospital of Qingdao University, Yantai, 264000 Shandong Province China

**Keywords:** Hepatocellular carcinoma, circTP63, miR-155-5p, ZBTB18, Cancer progression, Sponging

## Abstract

**Background:**

Hepatocellular carcinoma (HCC) is the leading cause of tumor-related death worldwide due to high morbidity and mortality, yet lacking effective biomarkers and therapies. Circular RNAs (circRNAs) are a group of non-coding RNAs that regulate gene expression through interacting with miRNAs, implicating in the tumorigenesis and progression. A novel circRNA, circTP63, was reported to be an oncogene in HCC. However, its role in HCC remains unclear.

**Methods:**

qRT-PCR was used to assess the mRNA levels of CircTP63 in 90 pairs of tumor and adjacent normal tissues from HCC patients, one human normal hepatic epithelial cell line and HCC cell lines. CCK-8, colony formation, transwell, and flow cytometry assays were performed to detect the cellular function of circTP63/miR-155-5p/ZBTB18 in HCC cells. HCC xenograft mice models were established to assess the in vivo effect of circTP63. Bioinformatic analysis, RNA pull-down and luciferase assays were used to determine the interaction among circTP63/miR-155-5p/ZBTB18.

**Results:**

circTP63 was significantly upregulated in HCC tissues and cell lines. High circTP63 expression is closely associated with the tumor stages, lymph node metastasis, and poor prognosis of HCC patients. Functionally, knockdown of circTP63 inhibited cell proliferation, migration, invasion, and promoted cell apoptosis of HCC. Meanwhile, overexpression of circTP63 enhanced HCC progression. Mechanically, circTP63 was a sponge of miR-155-5p to facilitate the ZBTB18 expression, and the ZBTB18 expression in HCC tissues was negatively associated with the survival rate of HCC patients. Furthermore, rescued assays revealed that the reduced tumor-promoting effect on HCC cells induced by knockdown of circTP63 can be reversed by miR-155-5p inhibitor or ZBTB18 overexpression.

**Conclusion:**

Our data highlight a critical circTP63-miR-155-5p-ZBTB18 regulatory network involved in the HCC progression, gaining mechanistic insights into the function of circRNAs in HCC progression, and providing effective biomarkers and therapeutic targets for HCC treatment.

## Background

Hepatocellular carcinoma (HCC) is the most common cause of cancer-related death worldwide in terms of high morbidity and mortality [1–3]. Despite great advances in the treatment of HCC, HCC is diagnosed at an advanced-stage accompanied by malignant proliferation in most patients, and the prognosis and survival rate of advanced-stage patients remains very poor [3]. Therefore, further research is needed to discover novel biomarkers and effective targets for HCC diagnosis and treatment.

Circular RNAs (circRNAs) are a novel class of non-coding RNAs, characterized by a covalently closed loop structure without any 5′ to 3′ polarity or a polyadenylated tail, and have no capacity to code protein [4, 5]. Many studies demonstrated that circRNAs regulate gene expression acting as competing endogenous RNA (ceRNAs), also serves as microRNAs (miRNAs) sponges, which interact with and inhibit miRNAs to reverse the suppression of target gene expression [6–8]. CircRNAs have been found to participate in various physiological and pathological processes, and act as potential biomarkers in many human cancers [9–12]. Especially, the roles of circRNAs in tumorigenesis are widely investigated. A novel circRNA, circTP63, was recently reported to be an oncogene in lung squamous cell carcinoma [13]. However, its role in other cancer types including HCC is unknown.

MiRNAs are a group of short non-coding RNAs with around 21 nucleotides, and repress target gene expression by binding to 3′ untranslated region (UTR) of mRNA [14]. The interactions between circRNAs and miRNAs play crucial roles in tumor development [6]. The significance of miRNAs in tumorigenesis as effective biomarkers are attracting more attention [14]. Some evidence suggested that miR-155-5p has been reported to be involved in HCC progression; Several certain long non-coding RNAs (lncRNAs) inhibit HCC development by sponging miR-155-5p [20]; miR-155-5p also could regulate the aggressiveness of HCC by regulating Wnt/β-catenin signaling or inhibiting PTEN via the PI3K/Akt pathway [21, 22]. Thus miR-155-5p would be a promising therapeutic target for HCC patients.

In this study, we aimed to explore the clinical significance and molecular mechanism of circTP63 in HCC. We found that significant upregulation of circTP63 in HCC tissues and cell lines. CircTP63 is associated with the tumor stages, lymph node metastasis, and poor prognosis of HCC patients. Of note, circTP63 promotes HCC progression by sponging miR-155-5p to elevate ZBTB18. Our study suggested that circTP63 exerts a tumor-promoting effect and it may be a candidate for diagnostic biomarker and therapeutic target of HCC. Our study will help us better understand the regulatory role of circRNAs in HCC progression, improving the diagnosis and therapies of HCC.

## Methods

### Clinical patient samples

Ninety pairs of HCC tumor and adjacent tissues were collected from patients admitted to the Department of Hepatobiliary Surgery, Yantai Mountain Hospital. All specimens were immediately frozen and stored in liquid nitrogen for further use. The detailed clinicopathological characteristics are described in Table 1. Inclusion criteria for HCC patients were curative hepatectomy performed between 2018 and 2019, diagnosed as HCC by two senior pathologists and no adjunctive treatment prior to the surgery. The exclusion criteria were other tumors, and incomplete clinical or prognostic data. For sampling, HCC tumor tissue was excised surgically while paired adjacent non-cancerous tissue was resected at the tumor over 3 cm away from the edge of the cancerous tissue. All samples were collected immediately after removal from the patients and snap-frozen in liquid nitrogen, followed stored at − 80 °C before further experiments. The study was approved by the ethical committee of the Department of Hepatobiliary Surgery, Yantai Mountain Hospital. Written informed consent was obtained from all patients.

**Table 1:** Correlation between circTP63 expression and clinicopathological features in HCC patients (n = 90)

Chinicopathological characteristics	Total	CircTP63 high expression (n = 45)	CircTP63 low expression (n = 45)	*X*^2^	*P* value
Gender					
Male	44	23	21	0.178	0.673
Female	46	22	24		
Age					
≤ 50	43	25	18	2.182	0.140
> 50	47	20	27		
Tumor size					
T1	26	7	19	12.492	0.006
T2	23	10	13		
T3	20	12	8		
T4	21	16	5		
Differentiation					
High	28	20	8	14.763	0.001
Moderate	29	17	12		
Poor	33	8	25		
Lymph node metastasis					
Positive	42	28	14	8.750	0.003
Negative	48	17	31		
TMN stages					
I	23	5	18	14.809	0.002
II	23	11	12		
III	23	14	9		
IV	19	15	4		

### Cell culture and transfection

One human normal hepatic epithelial cell line THLE-21 and six HCC cell lines (Hep3B, Huh7, MHCC97L, SK-hep1, SNU-387, SMMC7721) were purchased from Cell Bank of Type Culture Collection of the Chinese Academy of Sciences (Shanghai, China) and cultured in Dulbecco's modified Eagle's medium (DMEM) supplemented with 10% (v/v) fetal bovine serum (FBS) (Gibco, USA) in a humidified 37 °C incubator with 5% CO_2_. All cell lines used in our study have been authenticated using STR profiling. For circTP63 or ZBTB18 overexpression, the full-length sequence of circTP63 or ZBTB18 was cloned into pcDNA3.1 (Invitrogen, CA, USA) plasmid to generate pcDNA3.1-circTP63 or pcDNA3.1-ZBTB18. All small interfering RNAs (siRNAs), miR-155-5p mimics, inhibitor, and their NC negative controls were obtained from GeneCopoeia (Guangdong, China). Cell transfection was conducted with Lipofectamine 2000 Reagent (Invitrogen, CA, USA) as the manufacturer’s protocol. The siRNA sequences for transfection as follows:

si-circTP63#1, 5′-GCCAACAGUGAGGGGCCGU-3′;

si-circTP63#2, 5′-CAACAGUGAGGGGCCGUGAGA-3′;

siRNA control (si-NC) siRNAs 5′-UUCUCCGAACGUGUCACGU-3′.

### Quantitative reverse transcription-Polymerase Chain Reaction (qRT-PCR)

TRIzol reagent (Invitrogen, USA) was used to extract total RNA from the collected tumor samples and cells following the protocols. The cDNA was synthesized using a PrimeScript RT reagent kit (Takara, Japan). Gene expression was analyzed with SYBR Green Real-Time PCR Master Mixes (Thermo Fisher Scientific, USA) using an ABI 7900 Fast Thermal Cycler (Applied Biosystems; Thermo Fisher Scientific, USA), and GAPDH and U6 were served as an internal control to normalize mRNA and miRNA levels, respectively. The relative mRNA/miRNA expression was calculated using the 2^−ΔΔCt^ method. The primers for qRT-PCR were as follows:

GAPDH F: 5′-AAGGTGAAGGTCGGAGTCA-3′;

R: 5′-GGAAGATGGTGATGGGATTT-3′;

U6 F: 5′-CTCGCTTCGGCAGCACA-3′;

R: 5′-AACGCTTCACGAATTTGCGT-3′

circTP63 F: 5′-GCCCTCACTCCTACAACCATT-3′;

R: 5′-TTGTGTGCTGAGGAAGGTACT-3′;

miR-155-5p F: 5′-GAGGGTTAATGCTAATCGTGATAGG-3′;

R: 5′-GCACAGAATCAACACGACTCACTAT-3′;

### Bioinformatic analysis

StarBase 3.0 (http://starbase.sysu.edu.cn/) was used to predict the binding site of miR-155-5p within circTP63 and the targets of miR-155-5p as standard procedures.

### Cell counting kit-8 (CCK-8) assay

Cells at a density of 3 × 10^3^ per well were seeded into 96-well plates and incubated for 0, 24, 48 and 72 h. Next, 10 μL of CCK-8 solution (Dojindo, Japan) was added and incubated in the dark at 37 °C for another 1 h. The absorbance was detected using the microplate reader (Synergy H4 Hybrid Reader, BioTek, USA) at a wavelength of 450 nm.

### Colony formation assay

A total of 500 cells per well were plated into 6-well plates. After the cells were grown for about 10 days, cells were fixed with 4% paraformaldehyde and stained with crystal violet (Beyotime, China) for 30 min, and colonies were photographed under a Nikon Inverted Research Microscope Eclipse Ti microscope and quantified with imageJ4.

### Transwell assay

Transwell assay was performed to evaluate cell migration and invasion. After transfection, 1 × 10^5^ cells in 200 μL of FBS-free medium were seeded in the top chamber with a porous membrane with Matrigel solution (BD, USA) for invasion assay or non-coated membrane for migration assay, respectively. The bottom chamber was inserted into a 12-well filled with 800 μL complete medium containing 10% FBS. After 24 h of culturing at 37 °C, cells were removed from the upper surface of the membrane with cotton swabs, and cells on the lower surface of the chamber were fixed with 4% formaldehyde and stained with crystal violet (Beyotime, China). Five random fields per well were photographed and calculated using a Nikon Inverted Research Microscope Eclipse Ti microscope.

### Apoptosis assay

Transfected cells (1 × 10^6^ cells/well) were plated in 6-well plates. After treatment, cells were collected by centrifugation at 1500 rpm for 5 min and then incubated with 5 μL of FITC-conjugated Annexin V and 5 μL of PI for 20 min in the dark at 4 °C. The stained cells were detected by the BD FACS Aria II flow cytometer (BD Biosciences, USA).

### In vivo xenograft experiments

Six-week BALB/c nude female mice were used to perform xenograft experiments (n = 6/per group). All animal protocols were approved by the Institutional Animal Care and Use Committee at the Department of Hepatobiliary Surgery, Yantai Mountain Hospital. 1 × 10^7^ Hep3B cells transfected with the indicated siRNA using the in vivo transfection reagent, JetPEI (Polyplus Transfection, Illkirk, France) were subcutaneously injected into the flank. Mice were observed daily, and caliper measurements began once tumors became visible. The tumor volume was evaluated every 7 days via calipers, which were calculated using the following formula: Tumor volume (mm^3^) = (height) × (width)^2^/2. After 35 days, mice were sacrificed, and tumors were dissected and weighed. Tumor tissues were collected and snap frozen in liquid nitrogen and stored at − 80 °C for following analyses.

### Nuclear cytoplasmic fractionation

The cellular fraction was performed using NE-PER Nuclear and Cytoplasmic Extraction Reagents (ThermoFisher, USA) as the manufacturer's protocol. 5 × 10^6^ cells were re-suspended in buffer C (20 mM Tris–HCl pH 7.5, 75 mM NaCl, 5 mM MgCl_2_, 0.5% p/w sodium deoxycholate, 0.2% Triton, 1 mM DTT, 0.5% glycerol) supplemented with protease inhibitor cocktail (Sigma, USA) and 1 U/μL RNase inhibitor (Thermo Scientific, USA). After centrifugation, cytoplasmic lysate supernatants were carefully harvested. Then pelleted nuclei were washed extensively with PBS. Pelleted nuclei were resuspended in buffer N (10 mM Tris–HCl pH 8, 25 mM NaCl, 5 mM MgCl_2_, 1% p/w sodium deoxycholate, 1% Triton, 0.2% SDS, 1 mM DTT) supplemented with protease inhibitors and RNase inhibitors. After sonication, RNA from each portion was isolated for further analysis.

### Ribonuclease R (RNase R) treatment

3 μg total RNAs were incubated at 37 °C in 20 μL reactions containing 2 μL of 10 × RNase R Buffer (0.2 M Tris–HCl (pH 8.0), 1 mM MgCl_2_ and 1 M KCl, NaCl or LiCl) and 1 μL of RNase R (20 U/μL, Epicentre, USA) to remove linear RNAs and enrich circRNAs at 37 °C for 45 min. Controls were also conducted but the water was added instead of the RNase R enzyme. After RNase R treatment, the reactions were incubated at 70 °C for 10 min to deactivate the RNase R. The treated RNAs were used for qRT-PCR.

### Luciferase reporter assays

A total number of 3 × 10^4^ cells were plated in 24-well plates the day before transfection. Then cells were transfected with the wild-type or mutant circTP63/ZBTB18 reporter plasmids using Lipofectamine 2000 (Invitrogen, USA) as the manufacturer’s instructions. After 48 h of transfection, luciferase activities were measured using the Dual-Luciferase Reporter Assay System (Promega, USA).

### RNA pull-down

circTP63 and negative control Oligo were biotinylated by GenePharma Company (Shanghai, China). Next, they were transfected into HCC cells at 4 °C for 48 h. Cells were collected and incubated with Streptavidin-coupled magnetic beads (Invitrogen, USA) at 25 °C for 2 h. After cells were washed and eluted with buffer, the bound RNAs were quantified and analyzed by qRT-PCR.

### Western blot analysis

After treatment, cells were lysed in RIPA lysis buffer (Beyotime, China). Total protein concentration was subjected to the BCA Protein Assay (Beyotime, China). Next, proteins were separated on SDS-PAGE and transferred onto PVDF membranes (Millipore, USA), followed by blockade with 5% non-fat milk for 1 h at room temperature, and then incubated with corresponding primary antibodies (ZBTB18, ab118471, dilution 1:1000; GAPDH, ab181602, dilution 1:10,000) at 4 °C overnight. Then, the membranes were incubated with corresponding secondary antibodies. All these antibodies were purchased from Abcam (Cambridge, UK). An Immobilon Western Chemiluminescent HRP Substrate Kit (Millipore, USA) was used for protein bands detection. The protein bands were quantified with the ImageJ4.

### Statistical analysis

Statistical analysis was performed using Microsoft Office Excel and Graphpad7 software. Overall survival was analyzed by Kaplan–Meier and the log-rank test. The significance of difference was evaluated with Student’s t-test in two groups. One-way ANOVA was used in over two groups and different time points. *P* values less than 0.05 were considered significant (*, *P* < 0.05; **, *P* < 0.01). All data present the means ± SD. of three independent experiments.

## Results

### CircTP63 is upregulated in HCC and correlated with poor prognosis

**Fig. 1:**
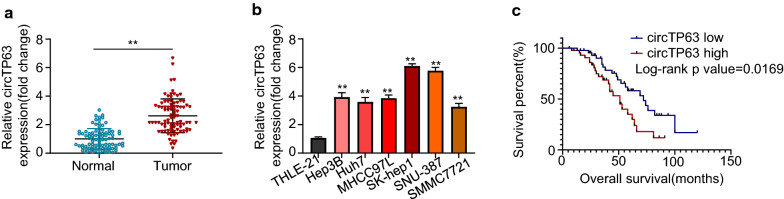
CircTP63 is upregulated in HCC and correlated with poor prognosis. **a** Relative mRNA expression levels of circTP63 in 90 pairs of HCC tissues and adjacent normal tissues were analyzed using qRT-PCR. **b** Relative mRNA expression levels of CircTP63 in one human normal hepatic epithelial cell line THLE-21 and six HCC cell lines (Hep3B, Huh7, MHCC97L, SK-hep1, SNU-387, SMMC7721). **c** Kaplan–Meier analysis was used to analyze the association between expression of circTP63 and the overall survival rate of HCC patients. Data are representative of three independent experiments and shown as mean ± SD., **P < 0.01

To explore the clinical implication of circTP63 in HCC, total RNA was collected from 90 pairs of HCC patient tumor tissues and matched adjacent normal tissue for qRT-PCR assay. We found that circTP63 was significantly upregulated in HCC tissues compared to that in normal tissues (Fig. 1a). Moreover, circTP63 expression levels in six HCC cell lines (Hep3B, Huh7, MHCC97L, SK-hep1, SNU-387, SMMC7721) were markedly increased relative to that in a normal human hepatic epithelial cell line THLE-21 (Fig. 1b). The expression level of circTP63 in SK-hep1 and SNU-387 cell lines was also confirmed by the RNase R experiment as shown in Additional file 1: Figure S1. Next all 90 patients were divided equally into circTP63 low expression (n = 45) and high expression (n = 45) groups from the medium value. Kaplan–Meier analyses demonstrated that patients with a high circTP63 level had a shorter overall survival rate relative to patients with low circTP63 expression (Fig. 1c). Further statistical analyses of clinicopathological features of HCC patients showed that upregulation of circTP63 was positively correlated to tumor-node-metastasis (TNM) stage, tumor differentiation, and lymph node metastasis (*P* < 0.05, Table 1), but there was no significant relationship between circTP63 expression and age or gender of patients. These results indicated that circTP63 promotes HCC progression.

### CircTP63 downregulation inhibits HCC cell proliferation, migration, invasion, and induces cell apoptosis

**Fig. 2:**
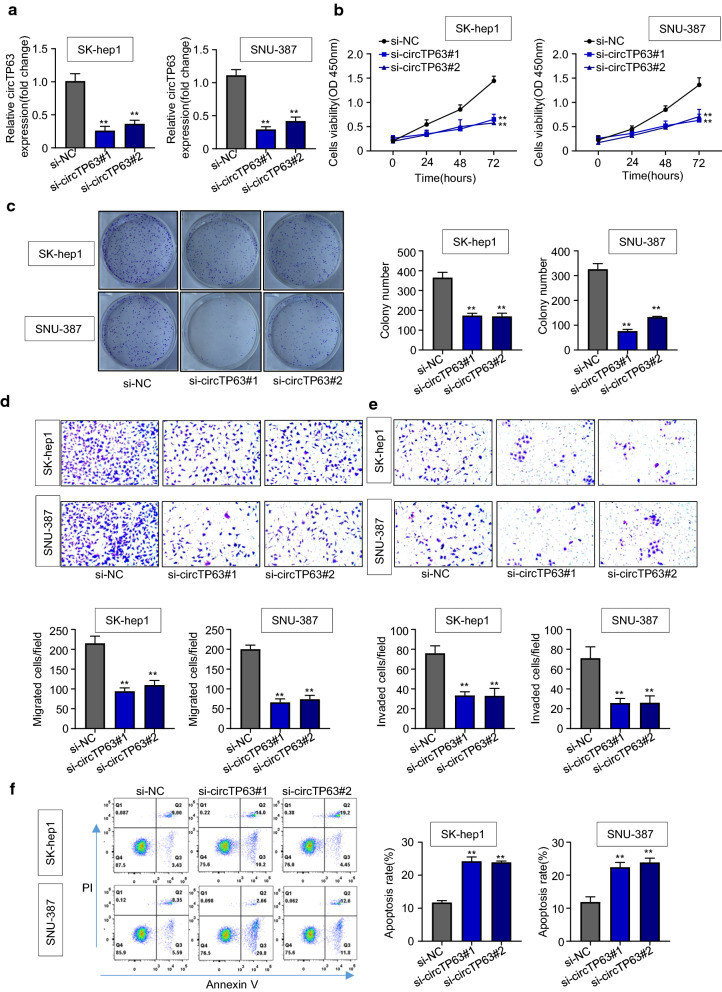
CircTP63 downregulation inhibits HCC cell proliferation, migration, invasion, and induces cell apoptosis. **a** qRT-PCR analysis of circTP63 mRNA expression in SK-hep1 and SNU-387 cells transfected with si-NC, si-circTP63#1 or si-circTP63#2. **b**, **c**. CCK8 and colony formation assays were conducted to assess the proliferation of SK-hep1 and SNU-387 cells transfected with si-NC, si-circTP63#1 or si-circTP63#2. **d**, **e** Transwell assays were used to assess the migration and invasion abilities of SK-hep1 and SNU-387 transfected with si-NC, si-circTP63#1 or si-circTP63#2. F. Annexin V/PI staining was used to assess the cell apoptosis of SK-hep1 and SNU-387 cells transfected with si-NC, si-circTP63#1 or si-circTP63#2 using flow cytometry. Data are representative of three independent experiments and shown as mean ± SD., **P < 0.01

To investigate the role of circTP63 in HCC progression, we knocked down circTP63 expression (si-circTP63#1 and si-circTP63#2) in SK-hep1 and SNU-387 cells which are circTP63-high expressed cell lines relative to other HCC cells (Fig. 2a). CCK-8 assay results demonstrated that knockdown of circTP63 substantially inhibited the proliferation of SK-hep1 and SNU-387 cells in a time-dependent manner (Fig. 2b). We also observed that the proliferation of cells transfected with si-circTP63 was significantly reduced compared with that of control cells. And there were no obvious changes in cellular shape and morphology (Additional file 1: Figure S2). Consistently, fewer colonies of SK-hep1 and SNU-387 cells were formed after circTP63 knockdown (Fig. 2c). We then assessed the migration and invasion capabilities of HCC cells after the circTP63 knockdown. Transwell assays results showed that circTP63 knockdown dramatically suppressed the migration and invasion of SK-hep1 and SNU-387 cells relative to that in the si-NC group (Fig. 2d, e). Furthermore, flow cytometry results showed that downregulated circTP63 promoted the apoptosis rate of SK-hep1 and SNU-387 cells (Fig. 2f). A rescue experiment for the circTP63 siRNA further confirmed the oncogenic role of circTP63 and excluded the off-target effect (Additional file 1: Figure S3). Collectively, our data suggested that circTP63 downregulation inhibits HCC cell proliferation, migration, invasion, and induces cell apoptosis.

### CircTP63 promotes HCC cell proliferation, migration, invasion, and suppresses cell apoptosis

**Fig. 3:**
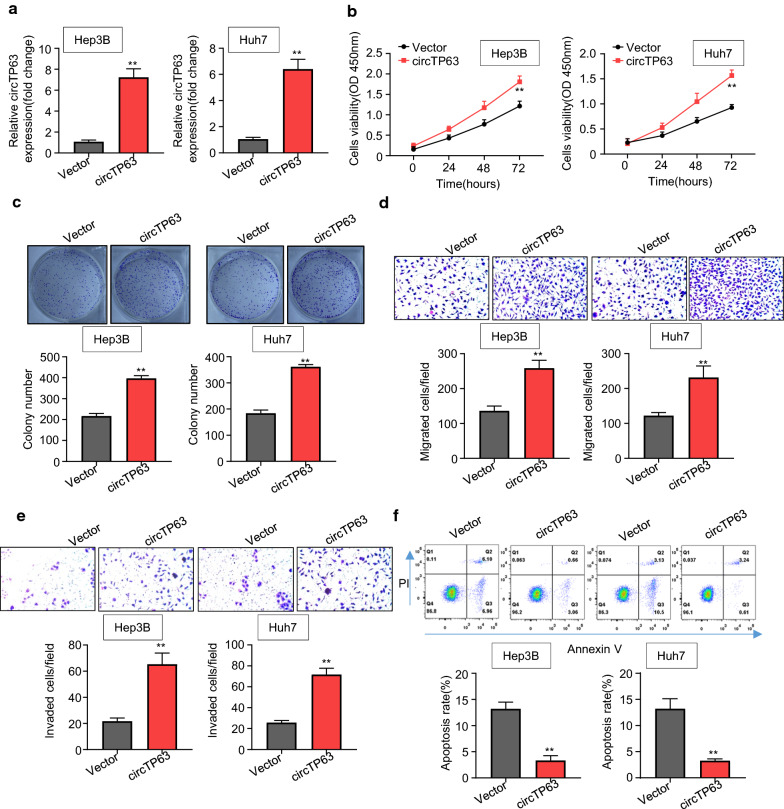
CircTP63 promotes HCC cell proliferation, migration, invasion, and suppresses cell apoptosis. **a** qRT-PCR analysis of circTP63 mRNA expression in Hep3B and Huh7 cells transfected with empty vector or pcDNA3.1-circTP63. **b**, **c** CCK8 and colony formation assays were used to measure the proliferation ability of Hep3B and Huh7 cells transfected with empty vector or pcDNA3.1-circTP63. **d**, **e** Transwell assays were used to determine the migration and invasion of Hep3B and Huh7 transfected with empty vector or pcDNA3.1-circTP63. **f** Annexin V/PI staining was used to assess the cell apoptosis of Hep3B and Huh7 cells transfected with empty vector or pcDNA3.1-circTP63 using flow cytometry. Data are from three independent experiments and presented as mean ± SD., **P < 0.01

We next overexpressed circTP63 in Hep3B and Huh7 cells, which are circTP63-low expressed cell lines (Fig. 3a). CCK-8 assays results showed that circTP63 upregulation remarkably promoted Hep3B and Huh7 cell proliferation in a time-dependent manner (Fig. 3b). More colonies were formed after circTP63 overexpression in Hep3B and Huh7 cells (Fig. 3c). Besides, the migration and invasion abilities of Hep3B and Huh7 cells were significantly increased after circTP63 overexpression (Fig. 3d, e). Flow cytometry results showed that increased circTP63 dramatically repressed the apoptosis rate of Hep3B and Huh7 cells (Fig. 3f). To assess the in vivo function of circTP63, we further established the Hep3B cells xenograft mice model. We observed that circTP63 overexpression led to an increment in volumes and weights of HCC tumors (Additional file 1: Figure S4). These results indicated that circTP63 promotes HCC cell proliferation, migration, invasion, and suppresses cell apoptosis.

### CircTP63 serves as a miR-155-5p sponge in HCC cells

**Fig. 4:**
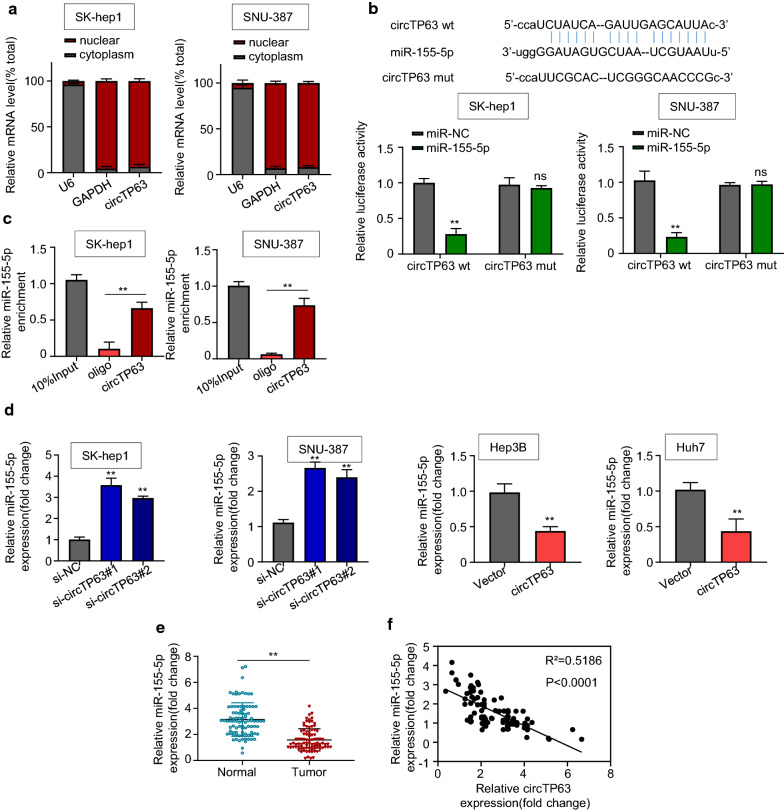
CircTP63 serves as a miR-155-5p sponge in HCC cells. **a** The mRNA levels of U6 (nuclear control), GAPDH (cytoplasmic control), and circTP63 were measured using qRT‐PCR in SK-hep1 and SNU-387 cells. **b** A predicted binding site of miR-155-5p within circTP63 by bioinformatic analysis using the StarBase 3.0 (http://starbase.sysu.edu.cn/). Luciferase activity was determined in SK-hep1 and SNU-387 cells after transfection with miR-155-5p mimic or miRNA negative control (miR-NC). **c**, **d** RNA pull-down and luciferase reporter assays were conducted to determine the interaction between circTP63 and miR-155-5p. E. Relative mRNA expression of miR-155-5p in HCC tissues and adjacent normal tissues was evaluated by qRT-PCR. **f** Pearson correlation analysis between circTP63 and miR-155-5p expressions in 90 pairs of HCC tissues. Data are representative of three independent experiments and shown as mean ± SD., **P < 0.01; ns, no significance

Many studies found that circRNAs serve as miRNA sponges to suppress their function [6–8, 10, 15]. qRT-PCR results showed that circTP63 was mainly located in the cytoplasm of SK-hep1 and SNU-387 cells (Fig. 4a), suggesting circTP63 may function as a sponge of miRNAs. To investigate the potential miRNA partners mediated by circTP63, we used the StarBase 3.0 database (http://starbase.sysu.edu.cn/). Results showed that miR-155-5p may bind to circTP63 (Fig. 4b). Luciferase reporter assays results further confirmed that the transfection of miR-155-5p mimics suppressed the luciferase activity of wide-type circTP63 (circTP63-wt), while the inhibitory effect was diminished by mutation of this binding motif (Fig. 4b). Furthermore, RNA pull-down assays results showed that the miR-155-5p expression was more enriched on biotin-labeled circTP63 probes (Fig. 4c), indicating that circTP63 can physically bind to miR-155-5p. Moreover, we observed that knockdown of circTP63 in SK-hep1 and SNU-387 cells led to the upregulation of miR-155-5p, whereas circTP63 overexpression in Hep3B and Huh7 cells contributed to the reduction of miR-155-5p (Fig. 4d). As shown in Fig. 4e, miR-155-5p was substantially downregulated in HCC tumor tissues by qRT-PCR assay in 90 pairs of human HCC samples (P < 0.001), and there was a significant negative association between circTP63 and miR-155-5p expression levels (Fig. 4f, Pearson *P* < 0.0001). Taken together, these results suggested that circTP63 directly targets miR-155-5p in HCC cells.

### ZBTB18 is a direct target gene of miR-155-5p in HCC

**Fig. 5:**
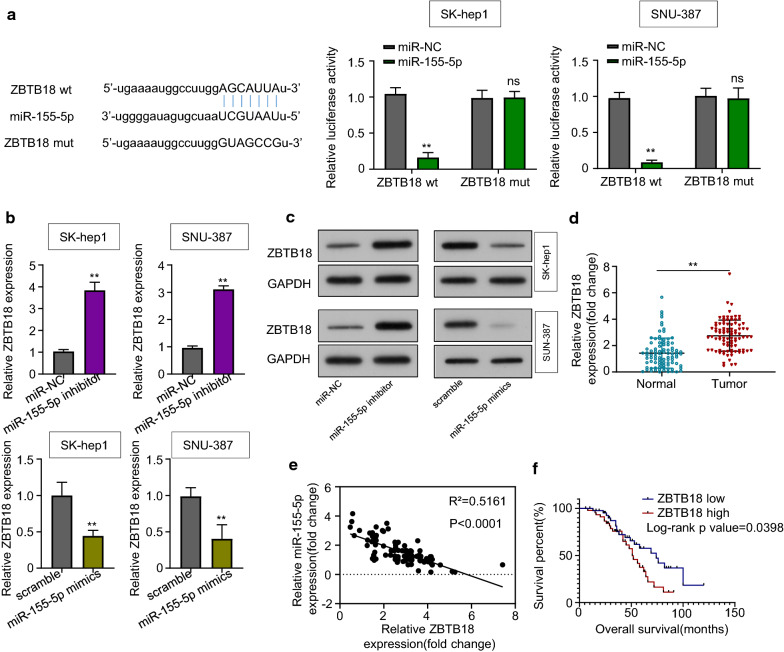
ZBTB18 is a direct target gene of miR-155-5p in HCC. **a** A predicted binding site of miR-155-5p within ZBTB18 by bioinformatic analysis using the StarBase 3.0 (http://starbase.sysu.edu.cn/). Luciferase activity was determined in SK-hep1 and SNU-387 cells after transfection with miR-155-5p mimic or miRNA negative control (miR-NC). **b**, **c** qRT-PCR and western blotting analysis were used to determine ZBTB18 expression in SK-hep1 and SNU-387 cells transfected with miR-NC/miR-155-5p inhibitor or scramble/miR-155-5p mimics. **d** Relative mRNA expression of ZBTB18 in HCC tissues and adjacent normal tissues was evaluated using qRT-PCR. **e** Pearson correlation analysis between miR-155-5p and ZBTB18 expressions in 90 pairs of HCC tissues. **f** Kaplan–Meier analysis was used to analyze the association between expression of ZBTB18 and the overall survival rate of HCC patients. Data are from three independent experiments and shown as mean ± SD., **P < 0.01; ns, no significance

To further investigate the mechanism of circTP63 and miR-155-5p, we also predicted the binding target of miR-155-5p using the StarBase 3.0 database. Bioinformatic results indicated some top-ranked genes targeted by miR-155-5p including ZBTB18. Among the potential candidate genes, ZBTB18 had the highest screening ranking score, suggesting that ZBTB18 would be a potential downstream target of miR-155-5p, which could bind to the 3′-UTR region of ZBTB18 (Fig. 5a). Luciferase assays confirmed that miR-155-5p could bind to ZBTB18, and suppressed ZBTB18 luciferase activity, but the inhibitory effect was reversed by mutation of this binding motif in SK-hep1 and SNU-387 cells (Fig. 5a). Besides, miR-155-5p inhibitor treatment increased both mRNA and protein levels of ZBTB18 in SK-hep1 and SNU-387 cells (Fig. 5b, c). In accordance with these results, the transfection of miR-155-5p mimics significantly reduced ZBTB18 expression (Fig. 5b, c), further confirming that miR-155-5p inhibits ZBTB18 expression. Finally, qRT-PCR analyses in 90 pairs of HCC clinical samples showed that the ZBTB18 mRNA level was much higher in HCC tissues compared with adjacent normal tissues (Fig. 5d). We also found that ZBTB18 and miR-155-5p expressions were negatively associated in HCC tissues (Fig. 5e, Pearson *P* < 0.0001). We then took the medium value of ZBTB18 in 90 pairs of HCC samples and divided them into ZBTB18 low expression (n = 45) and high expression (n = 45) groups. Kaplan–Meier analysis demonstrated that ZBTB18 low expression had a shorter overall survival rate of HCC patients (Fig. 5f).

### CircTP63 promotes HCC progression by regulation of the miR-155-5p/ZBTB18 axis

**Fig. 6:**
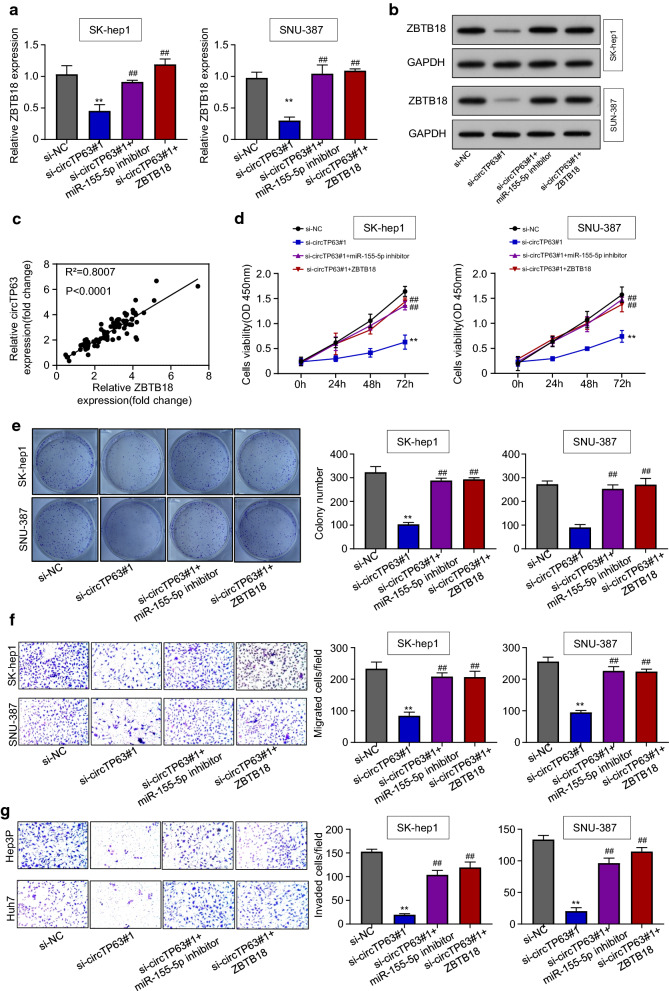
CircTP63 promotes HCC progression by regulation of the miR-155-5p/ZBTB18 axis. **a**, **b** mRNA and protein expression levels of ZBTB18 in SK-hep1 and SNU-387 cells transfected with si-NC, si-circTP63#1, si-circTP63#1 + miR-155-5p inhibitor or si-circTP63#1 + pcDNA3.1-ZBTB18 was evaluated using qRT-PCR and western blotting assays. **c** Pearson correlation analysis between circTP63 and ZBTB18 expressions in 90 pairs of HCC tissues. **d**, **e** CCK8 and colony formation assays were used to assess the proliferation of SK-hep1 and SNU-387 cells transfected with si-NC, si-circTP63#1, si-circTP63#1 + miR-155-5p inhibitor or si-circTP63#1 + pcDNA3.1-ZBTB18. **f**, **g** Transwell assays were used to assess the migration and invasion of SK-hep1 and SNU-387 cells transfected with si-NC, si-circTP63#1, si-circTP63#1 + miR-155-5p inhibitor or si-circTP63#1 + pcDNA3.1-ZBTB18. Data are representative of three independent experiments and shown as mean ± SD., **P < 0.01, compared to the si-NC group; ## *P* < 0.01, compared to the si-circTP63#1 group

To further determine whether circTP63 promotes HCC progression through regulation of the miR-155-5p/ZBTB18 axis, we treated miR-155-5p inhibitor or restored ZBTB18 expression in si-circTP63 HCC cells. qRT-PCR and western blotting results showed that knockdown of circTP63 downregulated both the mRNA and protein levels of ZBTB18, while the reduction was reversed by the treatment with miR-155-5p inhibitor or ZBTB18 overexpression in SK-hep1 and SNU-387 cells (Fig. 6a, b). Pearson analysis in 90 pairs of HCC samples revealed a significant correlation between circTP63 and ZBTB18 (Fig. 6c). Moreover, CCK-8 and colony formation results showed that miR-155-5p inhibitor or ZBTB18 overexpression rescued the anti-proliferative effect on SK-hep1 and SNU-387 transfected with si-circTP63 and formed more colonies (Fig. 6d, e). As shown in the transwell assays, we also observed that the migration and invasion abilities were restored after miR-155-5p inhibitor or ZBTB18 overexpression (Fig. 6f, g). Furthermore, we assessed the EMT-related protein levels of SK-hep1 and SNU-387 cells following miR-155-5p inhibitor or ZBTB18 overexpression. Western blotting experiment result demonstrated the miR-155-5p inhibitor treatment or ZBTB18 overexpression rescued the reduced expression of Twist, Snail, N-cadherin, Vimentin, P-FAK, and P-paxillin, as well as decreased the upregulated E-cadherin level in circTP63-knockdown HCC cells (Additional file 1: Figure S5). These data indicated that circTP63 promotes HCC progression by regulation of the miR-155-5p/ZBTB18 axis.

## Discussion

It is well known that circRNAs could function as endogenous miRNA sponges by interacting with miRNAs in the cytoplasm and regulating their function, which participates in tumor initiation and progression, thus becoming potential therapeutic targets and prognostic biomarkers for cancers [9–12]. A novel circRNA, circTP63, was recently identified to promote lung squamous cell carcinoma progression [13], but its functional role and molecular mechanism in HCC remains unknown. Our results showed that the expression of circTP63 was significantly upregulated in HCC tissues and cell lines, which was closely related to clinicopathological features and shorter survival of HCC patients. The knockdown of circTP63 inhibited HCC cell proliferation, migration, invasion, and promoted cell apoptosis, while overexpression of circTP63 had the reverse phenotypic effect on HCC cells. These results suggested that circTP63 plays an oncogenic role in HCC progression.

More recently, the involvement of miRNAs in tumorigenesis and their significance as effective biomarkers are also becoming increasingly appreciated [14]. MiR-155-5p has been found to regulate the development of various cancer types, including human epithelial ovarian cancer [16], cervical cancer [17, 18], and colon cancer [19]. It has been reported that some certain long non-coding RNAs (lncRNAs) suppress HCC development by sponging miR-155-5p [20]; miR-155-5p modulated the malignancy of HCC by regulating Wnt/β-catenin signaling or suppressing PTEN through the PI3K/Akt pathway [21, 22]; therefore, miR-155-5p can be a promising therapeutic target for HCC patients.

**Fig. 7:**
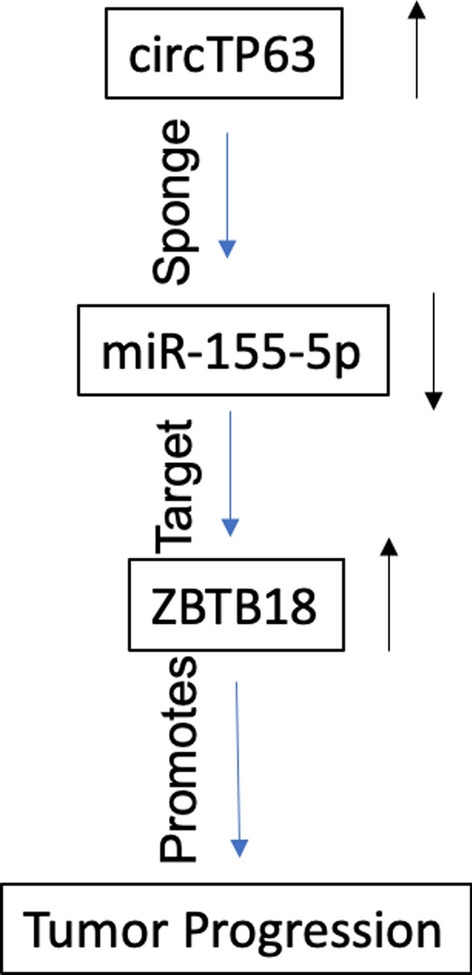
A schematic diagram that circTP63 promotes HCC progression by regulation of the miR-155-5p/ZBTB18 axis

It has been reported that Zinc Finger and Broad complex, Tramtrack, Bric_a brac (BTB) Domain Containing 18 (ZBTB18/ZNF238) is a zinc finger transcriptional repressor that belongs to the BTB or poxvirus and zing finger (POZ)-zinc finger (BTB/POZ-ZF) protein family involving in brain development and neuronal differentiation [23, 24]; ZBTB18 serves as a tumor suppressor in glioblastoma [25, 26]. However, the underlying mechanism of ZBTB18 downregulation in other cancer types also remains undefined. In our study, circTP63 was confirmed to be largely distributed in the cytoplasm. Bioinformatic analysis (StarBase 3.0) predicted that miR-155-5p was the potential downstream target of circTP63. The results of RNA pull-down and luciferase assays further confirmed their direct interaction in HCC. Mechanically, circTP63 obviously accelerated the HCC proliferation, migration, and invasion, and decreased cell apoptosis by facilitating ZBTB18 expression via sponging miR-155-5p in HCC, and the ZBTB18 expression in HCC tissues was negatively associated with the survival rate of HCC patients, implying that ZBTB1 plays a tumor-promoting effect on HCC progression. Furthermore, knockdown of circTP63 could reduce the oncogenic effect on HCC, which was partially rescued by miR-155-5p inhibitor or ZBTB18 overexpression. One potential limitation of the study is that we should also test the effect of circTP63 deletion in the mice xenograft model. Therefore, we are unable to confirm the potential in vivo efficacy of circTP53 inhibition exerted during the HCC progression. However, based on our cellular and clinical findings, we confirmed that circTP63 can promote HCC by sponging miR-155-5p and upregulating ZBTB18 (Fig. 7). We would determine the in vivo role of circTP63 in further investigations. As a potential target, we can conduct a high-throughput screening for small molecules to identify potential inhibitors of the circTP63/miR-155-5p/ZBTB18 axis.

In sum, our data firstly determined the expression, clinical implication, and mechanism of circTP63/miR-155-5p/ZBTB18 in HCC, and revealed that circTP63 facilitates HCC progression by regulating the miR-155-5p/ZBTB18 axis, providing promising clinical biomarkers and a therapeutic strategy for the treatment of HCC.

## Conclusion

We concluded that circTP63 promotes HCC progression by sponging miR-155-5p and upregulating ZBTB18, which provides a novel clinical biomarker and therapeutic target for HCC treatment.

## Supplementary Information

**Additional file 1: Figure S1.** For the circTP63 mRNA level in SK-hep1 and SUN-387 after treatment by RNase R in Fig. 1. **Figure S2.** For the morphological analysis of SK-hep1 and SUN-387 cells after si-circTP63 in Fig. 2. **Figure S3.** For the rescue experiment for the circTP63 siRNAs in RT-qPCR (A), Cell viability (B), Cell migration and invasion (C), and Colony formation (D) assays. **Figure S4.** For Tumor volumes and weights of Hep3B cells with circTP63 overexpression xenograft nude models. **Figure S5.** For the EMT biomarker analysis in SK-hep1 and SUN-387 cells transfected with si-NC, si-circTP63#1, si-circTP63#1 + miR-155-5p inhibitor or si-circTP63#1 + pcDNA3.1-ZBTB18.

## Data Availability

The datasets used and/or analyzed during the current study are available from the corresponding author on reasonable request.
