# Supplementary material for: CircTP63 promotes hepatocellular carcinoma progression by sponging miR-155-5p and upregulating ZBTB18
Source: Cancer Cell Int. 2021 Mar 8;21:156. doi: 10.1186/s12935-021-01753-x (PMC7938576; doi:10.1186/s12935-021-01753-x)

**Figure S1** for the circTP63 mRNA level in SK-hep1 and SUN-387 after treatment by RNase R in Figure 1.

**
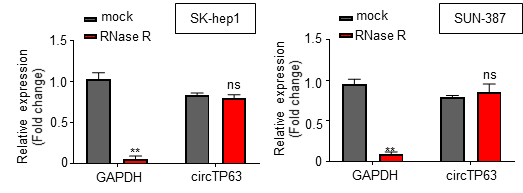
**

**Figure S2** for the morphological analysis of SK-hep1 and SUN-387 cells after si-circTP63 in Figure 2.


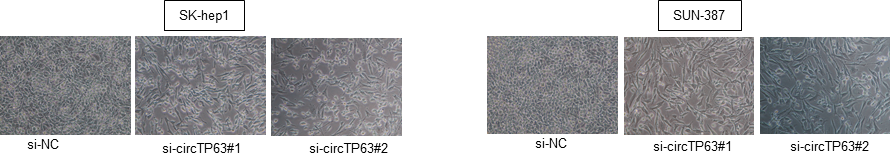


**Figure S3** for the rescue experiment for the circTP63 siRNAs in RT-qPCR (A), Cell viability (B), Cell migration and invasion (C), and Colony formation (D) assays.

**
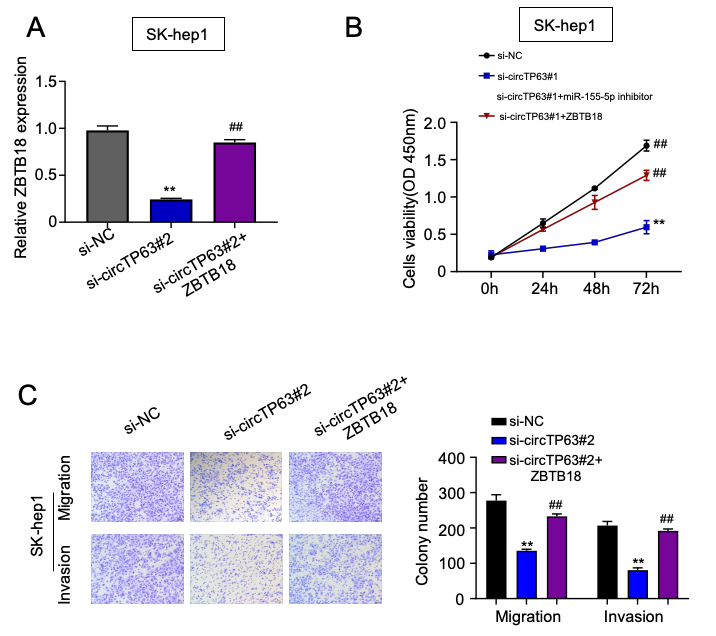
**

**Figure S4** for Tumor volumes and weights of Hep3B cells with circTP63 overexpression xenograft nude models.


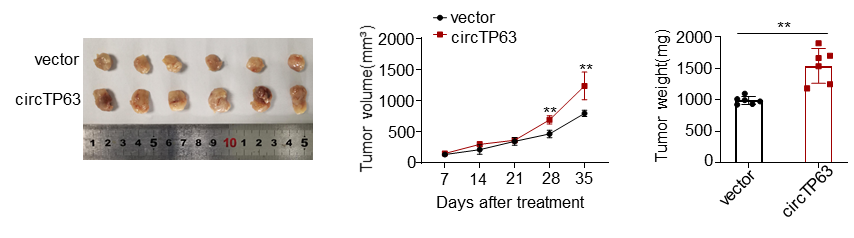


**Figure S5** for the EMT biomarker analysis in SK-hep1 and SUN-387 cells transfected with si-NC, si-circTP63#1, si-circTP63#1 + miR-155-5p inhibitor or si-circTP63#1 + pcDNA3.1-ZBTB18.


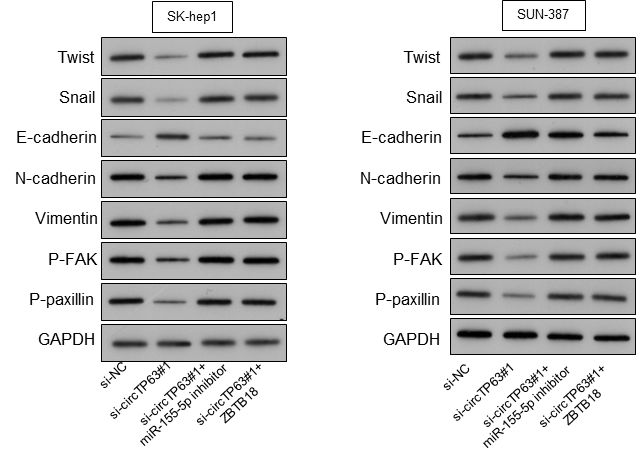

Supplement: Supplementary file 1 — Additional file 1: Figure S1. For the circTP63 mRNA level in SK-hep1 and SUN-387 after treatment by RNase R in Fig. 1. Figure S2. For the morphological analysis of SK-hep1 and SUN-387 cells after si-circTP63 in Fig. 2. Figure S3. For the rescue experiment for the circTP63 siRNAs in RT-qPCR (A), Cell viability (B), Cell migration and invasion (C), and Colony formation (D) assays. Figure S4. For Tumor volumes and weights of Hep3B cells with circTP63 overexpression xenograft nude models. Figure S5. For the EMT biomarker analysis in SK-hep1 and SUN-387 cells transfected with si-NC, si-circTP63#1, si-circTP63#1 + miR-155-5p inhibitor or si-circTP63#1 + pcDNA3.1-ZBTB18. [file 12935_2021_1753_MOESM1_ESM.docx]
